# Multi-omics strategy reveals potential role of antimicrobial resistance and virulence factor genes responsible for Simmental diarrheic calves caused by *Escherichia coli*

**DOI:** 10.1128/msystems.01348-23

**Published:** 2024-05-14

**Authors:** Zhihai Shi, Yali Lan, Yazhou Wang, Xiangzhou Yan, Xiaoya Ma, Faiz-ul Hassan, Hossam E. Rushdi, Zhaoxue Xu, Wenjia Wang, Tingxian Deng

**Affiliations:** 1 Institute of Animal Husbandry and Veterinary Medicine, Henan Academy of Agricultural Sciences, Zhengzhou, China; 2 Guangxi Provincial Key Laboratory of Buffalo Genetics, Breeding and Reproduction Technology, Buffalo Research Institute, Chinese Academy of Agricultural Sciences, Nanning, China; 3 Institute of Animal and Dairy Sciences, University of Agriculture, Faisalabad, Pakistan; 4 Department of Animal Production, Faculty of Agriculture, Cairo University, Giza, Egypt; 5 College of Veterinary Medicine, Henan University of Animal Husbandry and Economy, Zhengzhou, Henan, China; University of California, Davis, Davis, California, USA

**Keywords:** antibiotic resistance genes, diarrhea, metagenome, metabolomics, virulence factor genes

## Abstract

*Escherichia coli* (*E. coli*) is reported to be an important pathogen associated with calf diarrhea. Antibiotic resistance genes (ARGs) and virulence factor genes (VFGs) pose a considerable threat to both animal and human health. However, little is known about the characterization of ARGs and VFGs presented in the gut microbiota of diarrheic calves caused by *E. coli*. In this study, we used multi-omics strategy to analyze the ARG and VFG profiles of Simmental calves with diarrhea caused by *E. coli* K99. We found that gut bacterial composition and their microbiome metabolic functions varied greatly in diarrheic calves compared to healthy calves. In total, 175 ARGs were identified, and diarrheal calves showed a significantly higher diversity and abundance of ARGs than healthy calves. Simmental calves with diarrhea showed higher association of VFGs with pili function, curli assembly, and ferrienterobactin transport of *E. coli*. Co-occurrence patterns based on Pearson correlation analysis revealed that *E. coli* had a highly significant (*P* < 0.0001) correlation coefficient (>0.8) with 16 ARGs and 7 VFGs. Metabolomics analysis showed that differentially expressed metabolites in Simmental calves with diarrhea displayed a high correlation with the aforementioned ARGs and VFGs. Phylotype analysis of *E. coli* genomes showed that the predominant phylogroup B1 in diarrheic Simmental calves was associated with 10 ARGs and 3 VFGs. These findings provide an overview of the diversity and abundance of the gut microbiota in diarrheic calves caused by *E. coli* and pave the way for further studies on the mechanisms of antibiotic resistance and virulence in the calves affected with diarrhea.

**IMPORTANCE:**

Simmental is a well-recognized beef cattle breed worldwide. They also suffer significant economic losses due to diarrhea. In this study, fecal metagenomic analysis was applied to characterize the antibiotic resistance gene (ARG) and virulence factor gene (VFG) profiles of diarrheic Simmental calves. We identified key ARGs and VFGs correlated with *Escherichia coli* isolated from Simmental calves. Additionally, metabolomics analysis showed that differentially expressed metabolites in Simmental calves with diarrhea displayed a high correlation with the aforementioned ARGs and VFGs. Our findings provide an insight into the diversity and abundance of the gut microbiota in diarrheic calves caused by *Escherichia coli* and pave the way for further studies on the mechanisms of antibiotic resistance and virulence in the diarrheal calves from cattle hosts.

## INTRODUCTION

Calf diarrhea is a common disease that leads to enormous economic and production losses for cattle producers worldwide. It is well established that the diarrhea incidence is strongly associated with gut microbiota (1–3). Gut microbiota plays vital roles in shaping key aspects of postnatal life, including the development of the gut and immune system in newly born calves (4), influencing the host’s energy balance (5), and preventing colonization of foreign pathogens (6). Therefore, understanding the composition and functional properties of gut microbiota is imperative to control the occurrence and adverse effects of neonatal calf diarrhea.

Gut microbiome in ruminants consists of different types of microorganisms that produce thousands of bioactive compounds and coexists harmoniously in the habitat of the host gut (7–9). Diversity of gut microbiota is an indicator of the effective microbial ecosystem and the host’s health. Characterization of the diversity of gut microbiota is imperative for a better understanding of gut ecology and its physiological manifestations. To date, 16S rRNA gene sequencing and shotgun metagenomic sequencing are the two swordsmen for detecting microorganisms and assessing microbial diversity. The 16S rRNA gene sequencing has been widely used to identify microbiota and its diversity analysis in different livestock species, such as cattle (10), goats (11), sheep (12), and buffalo (13). In contrast, the shotgun metagenomic sequencing allows researchers to sequence all given genomic DNA from a complex sample, unlike 16S rRNA gene sequencing which only targets 16S rRNA genes. Hence, shotgun metagenomic sequencing can further explore the functional characteristics of microorganisms. Moreover, shotgun metagenomic sequencing has also been widely used for investigating microbial diversity in different farm animal species, including cattle (14), goats (15), sheep (16), and buffalo (17). Recently, the use of 16S rRNA gene sequencing of the fecal microbiota of calves revealed that microbial metabolism-related genes, such as starch and sucrose metabolism, sphingolipid metabolism, alanine aspartate, and glutamate metabolism, were significantly altered in diarrheal calves (18). However, the application of shotgun metagenomic sequencing for the identification of microbiota associated with calf diarrhea is still limited.

Numerous studies have characterized microbial diversity using metagenomic analysis; however, little is known about the identification of antibiotic resistance genes (ARGs) and virulence factor genes (VFGs) in the fecal microbiota of animals using the metagenomic approach. The ARGs and VFGs are well known to pose substantial health risks to both animals and humans. While ARGs contribute to resistance to antibiotics, VFGs facilitate infection and survival in adverse environments (19). It is documented that antimicrobial resistance levels vary greatly across countries, regions, and even herds or breeds (20–23). Therefore, the issues of antibiotic resistance and virulence are attracting wide concern from the scientific community. Identification of the ARGs and VFGs is imperative to better understand pathogenesis and devise strategies to control disease and antibiotic resistance. Although many ARGs and VFGs have been identified for microbes found in the environment, these factors are poorly understood in the gut microbiome of ruminants.

*Escherichia coli* (*E. coli*) is an important pathogenic bacterium associated with diarrhea. According to the host or organ tropism, *E. coli* genomes were categorized into different pathotypes, such as extraintestinal pathogenic *E. coli*, intestinal pathogenic *E. coli*, and uropathogenic *E. coli* (24). Additionally, *E. coli* genomes were also grouped into the diarrhea-associated hemolytic *E. coli* and enterohemorrhagic *E. coli* (EHEC) based on the disease conditions, as well as Shiga toxin-producing *E. coli* (STEC) based on the virulence factors (25). For instance, several antibacterial resistance genes involved in canine diarrhea have been identified in different strains of *E. coli,* including EHEC, STEC, and necrotoxic *E. coli* (26). Eltai et al. (27) reported that diarrhea incidence caused by *E. coli* (DEC) strains had a high rate of antimicrobial resistance, and the atypical enteropathogenic *E.coli* and enteroaggregative *E. coli* were the primary etiological agents of diarrhea in children among DEC pathotypes. It is well documented that *E. coli* can be commensal or pathogenic because of its high genetic plasticity (28).The phylogroups of *E. coli* have been described (A, B1, B2, C, D, E, and F) on the basis of multilocus sequence typing (MLST) profiles (29, 30). Moreover, *E. coli* species are currently divided into 183 O-serogroups (lipopolysaccharide) and 53 H-types (flagellar antigen) (31). In epidemiological studies, knowledge of the O-serogroups can help identify pathogenic lineages, classify *E. coli* for epidemiological studies, and identify outbreaks of diseases (32). Importantly, the relationship between antibiotic resistance and serogroups and virulence factors of *E. coli* was discovered in different disease conditions, such as children with septicemia (33), patients with urinary tract infection (34), and hospitalized patients (35). Nevertheless, similar studies on calf diarrhea are relatively limited.

In China, calf diarrhea is a common disease in cattle industry that causes huge morbidity and mortality losses. Reducing the occurrence of calf diarrhea is also a key health challenge faced by the Chinese cattle industry. Despite the importance of the gut microbiota in modulating host health, little is known regarding changes in the gut microbiota, ARGs, and VFGs and their potential associations with pathological status in different calf breeds affected by diarrhea. To devise effective strategies to control calf diarrhea, it is essential to better understand the gut microbiome, ARGs, and VFGs. In the present study, samples from calves suffering from diarrhea were taken from a Simmental cattle farm located in Henan province of China. In this regard, the aim of the present study was to characterize the changes in the gut microbiome and resistome of calves affected by diarrhea from Chinese Simmental beef cattle breed. Furthermore, we compared the differences in the relative abundances of ARGs and VFGs between the diarrheic calves due to *E. coli* and the healthy individuals. We believed that our potential findings would provide crucial insights into the gut microbiome of neonatal calves affected by diarrhea and pave the way for studies aimed at elucidating the antibiotic resistance and virulence mechanisms in diarrhea.

## MATERIALS AND METHODS

### Experimental design

A total of 257 fecal samples were collected from Simmental calves with diarrhea reared at a cattle farm attached to the Institute of Animal Husbandry and Veterinary Medicine, Henan Academy of Agricultural Sciences (Luoyang, China). Pathogen detection for all fecal samples was performed as previously described (36), and then the IDEXX Rota-Corona-K99 Ag Test was used to confirm the incidence of diarrhea. In briefly, fecal samples were fully resuspended in phosphate-buffered saline (1:10 [wt/vol]) and centrifuged at 10,000 × *g* for 10 min at 4°C. Next, viral RNA and DNA were extracted from 200 µL of the fecal suspension using the TaKaRa MiniBEST Viral RNA/DNA Extraction Kit Ver. 5.0 (TaKaRa Bio Inc.) according to the manufacturer’s instructions; another 200 µL of a suspension was used directly for the extraction of bacteria and protozoa nucleic acids with a QIAamp Fast DNA Stool Mini Kit (QIAGEN, Hilden, Germany). Subsequently, the multiplex PCR assay was used in the present study. PCR reaction mixture was composed of 10 µL of 2× Mix (TaKaRa Bio Inc.), 1.0 µL of each primer (10 µM), and 20 ng of template DNA in a total volume of 20 µL. All the PCR reactions were performed using a Thermal Cycler Dice Model TP600 (TaKaRa Bio Inc.). The detected pathogens consisted of the group A rotavirus, group B rotavirus, group C rotavirus, bovine coronavirus, bovine torovirus, bovine norovirus, bovine enteric Nebraska-like calicivirus, bovine nebovirus, bovine viral diarrhea virus, *Clostridium perfringens, Salmonella enterica, Salmonella enterica* Typhimurium*, Escherichia coli,* and *Eimeria zuernii*. Finally, 30 fecal samples, including 15 diarrheal calves with only positive K99 and 15 healthy individuals, were selected for shotgun metagenomic sequencing and non-targeted metabolomic sequencing. While not all calves were kept in the same space, a physical space was created for them to eat and interact with other calves during the experimental period (from calf birth to 18 days old). No routine treatments were performed except navel disinfection.

All selected calves were given the same colostrum at 8% of body weight within 2 h after birth, then antibiotic-free processed milk at 10% of BW was provided twice a day (06:00, 18:00) during their first 7 days of age. No waste milk (milk from cows treated with antibiotics or from cows with clinical mastitis) was added to the good milk fed to all calves. All feeding procedures were carried out by a professional operator using nipple buckets. Calves were gradually removed from bottle feeding and encouraged to drink milk from the buckets. Water was available *ad libitum* during the study period. All calves were provided *ad libitum* access to a calf starter (20% CP, Purina) from the seventh day of life until the end of the experiment.

The status and severity of diarrhea in the neonatal calves were assessed through a previously described procedure (37). Briefly, fecal scoring was recorded according to a standard scoring procedure (0 = normal feces; 1 = semi-formed feces; 2 = loose feces; 3 = watery feces). Individual calves were sampled before receiving antibiotics. Fecal samples for each individual were collected by the rectal palpation method while wearing clean disposable latex gloves and then stored at −80°C until DNA extraction. Details regarding sample collection from calves are summarized in Table S1 at https://doi.org/10.7910/DVN/UKE1CC.

### DNA extraction and shotgun metagenomic sequencing

Total DNA was isolated from 200 mg of fecal samples using the QIAamp PowerFecal DNA Kit (Qiagen, Maryland, USA) following the manufacturer’s instructions. DNA concentration and purity were measured using a Qubit spectrophotometer (Nanodrop Technologies Inc., DE, USA) and 1.5% agarose gel electrophoresis, respectively. Paired-end library construction for each sample was performed by using the TruSeqTM DNA Sample Prep Kit (Illumina Inc., CA, USA) according to the manufacturer’s instructions. Overall, genomic DNA samples were first fragmented to an average size of 350 bp using Covaris M220 (Gene Company Limited, Guangzhou, China). Then, the obtained fragments were end-repaired, A-tailed, and further ligated with adapters, as after that those ligated fragments were amplified by PCR, size selected, and purified. Finally, paired-end sequencing was carried out using Illumina NovaSeq System platform (Illumina Inc., CA, USA).

### Metagenomics analysis

Raw sequence data for each sample were converted and filtered into clean data by the readfq toolkit ver8 (https://github.com/cjfields/readfq). The filtration criteria applied were as follows: (i) remove reads with low-quality bases that exceed 40 bp; (ii) exclude reads with N bases reaching 10 bp; and (iii) remove reads whose overlaps with adapters exceed 15 bp. In addition, clean data were mapped into the bovine reference genome (*ARS-UCD1.2*) using the Bowtie ver2.2.4 software (38), and any hits associated with clean data and their mated reads were also removed.

The clean data were used for assembling analysis using the MEGAHIT ver1.0.4 software (39) with default settings. The scaftigs with a length ≥500 bp were used to predict the genes using MetaGeneMark ver2.10 software (40). The CD-HIT ver4.5.8 software (41) with the parameters (-c 0.95 -aS 0.9) was utilized for clustering genes from each sample. Genes with reads >2 in each sample were retained and defined as the final non-redundant gene catalog in this study. Quantification of unique genes in each sample was performed using Salmon ver1.9.0 software (42). The relative abundances of genes was presented with transcripts per kilobase of exon model per million mapped reads (TPM) based on the following equation:


$$TPM=\frac{N_{i}}{L_{i}}\left(\frac{1}{\sum_{j}\frac{N_{j}}{L_{j}}}\right)10^{6}$$

where *N_i_* is the number of reads mapped to the *i*th gene, *L_i_* is the total length of an exon in the *j*th gene. Non-redundant gene sequences were taxonomically assigned using DIAMOND ver.0.9.9 software (43) against the NCBI NR database ver. 2018.01.02. Taxonomic profiles were determined at the phylum, class, order, family, genus, and species levels, and the relative abundances at those taxonomic ranks were analyzed using MetaPhlAn ver.3.0 software (44). Taxonomic and phylogenetic trees of the gut microbiome from the two studied groups were generated by GraPhlAn ver1.1.3 software (45). The amino acid sequences of non-redundant genes were functionally annotated using Diamond ver.0.9.9 software (43) against the Kyoto Encyclopedia of Genes and Genomes (KEGG; version 2018-01-01), carbohydrate-active enzymes (CAZy; version 2018-01-01), and EggNOG (v4.5) databases.

### Non-targeted metabolomic analysis of fecal samples

The fecal samples from 30 calves were collected. The sample processing and sequencing were performed as described by our previous study (46). Here, a total of 14 diarrhea calves and 14 healthy individuals met the requirements for non-targeted metabolomic sequencing and were used for further analysis. The Compound Discoverer 3.1 (Thermo Fisher Scientific, Waltham, MA, USA) was used to process the raw data. The normalized data were used to predict the molecular formula based on additive ions, molecular ion peaks, and fragment ions. The qualitative and relative quantitative results of peaks were performed by the mzCloud (https://www.mzcloud.org/), mzVault, and MassList databases. Additionally, three databases including KEGG, Human Metabolome Database, and LIPID MAPS were used for metabolite annotation. Identification of differentially expressed metabolites (DEMs) was performed using univariate data analysis (*t*-test) with the following thresholds: projection (VIP) > 1, *P*-value < 0.05, and fold change > 2. KEGG analysis for DEMs was performed using the MetaboAnalyst (V5.0) (47).

### Identification of antibiotic resistance genes and virulence factors in metagenomes

Relative abundances of ARGs and VFGs in the gut microbiome were quantified using abricate ver.1.0.0 software (48) against the Comprehensive Antibiotic Resistance Database (CRAD; ver.2.0.1) and virulence factor database (VFDB). To determine the relative abundances of both ARGs and VFGs at the species level, we summed up the TPMs of those ARGs or VFGs under each specific taxonomic group.

### Co-occurrence network analysis for ARGs, VFGs, microbial taxa, and metabolites

To investigate the co-occurrence pattern of microbial communities, resistomes, and metabolites, a correlation matrix was constructed by calculating the pairwise Pearson correlation with the “psych” R package. Both correlation coefficient of >0.8 and adjusted *P*-value of <0.05 were defined as the threshold levels. Finally, Cytoscape ver.3.10.1 software (49) was applied to convert the resulting correlation matrix into an associated network.

### Phylotyping and serotyping for *Escherichia coli*

To obtain the sequence of *E. coli* genomes for each individual, the sequence of *E. coli* was first extracted from the NT database and used as the reference. All the assembled sequences for each sample were mapped into the reference genome using mummer ver. 3.23 (50) with the following parameters: nucmer --maxmatch --nosimplify -g 5000 c 200 L 500 show-coords -rlo -L 500. Only the contigs for each sample were defined as the sequence of *E. coli* genomes with the threshold of similarity > 0.95 and coverage > 0.90. Moreover, we downloaded a total of 10,061 *E. coli* genomes from NCBI as of 24 November 2023, with the criteria that each assembly has (i) >200 scaffolds/contigs, (ii) GC percentage > 0.47, (iii) genome size > 2 Mb, and (iv) coding DNA sequence > 2,000 bp. The detailed information on the *E. coli* genomes is listed in Table S2 at https://doi.org/10.7910/DVN/UKE1CC. Sequence types (ST) of *E. coli* genomes were identified using *in silico* MLST ver.2.23.0 (51) software based on PubMLST database (https://pubmlst.org). Phylogroup typing of all *E. coli* genomes was performed using EzClermont ver.0.6.3 (52) software. The *in silico* prediction of *E. coli* serotype was identified using ECTyper ver. 1.0.0 (53) software. Additionally, the abricate ver.1.0.0 (54) software was applied to predict the ARGs and VFGs for *E. coli* genomes against the CRAD (identity = 0.98 and coverage = 1.0) and VFDB (identity > 0.98 and coverage = 1.0) database, respectively.

### Statistical analysis

One-way analysis of variance was performed using GraphPad Prism ver.10.0.3 (*P* < 0.05). Differences in relative abundances of taxa, functional genes, metabolic pathways, ARGs, and VFGs between different comparisons were identified using linear discriminant analysis effect size (LEfSe) or Wilcoxon’s rank-sum test. LEfSe analysis was performed by using the LEfSe ver.1.1.2 software. Significance testing of the principal coordinates analysis (PCoA) was performed based on the Bray-Curtis distances using the “vegan” R package (55). The analysis of similarity (ANOSIM) was applied to determine the difference in abundances of gut microbiome or ARGs between DSM and HSM groups. ANOSIM analysis was performed by using the “vegan” R package. The Benjamini and Hochberg false discovery rate was used to correct the *P*-value. Simpson, Shannon, and Chao indexes of the gut microbiota, ARGs and VFGs between comparison groups were performed to evaluate their diversity using the “vegan” R package. All plots were mainly visualized by ggplot2 packages (56).

## RESULTS

### Demographic parameters of the study calves

In this study, 30 Simmental calves were used. Details regarding their characteristics are presented in Table S3 at https://doi.org/10.7910/DVN/UKE1CC. Age, sex, and temperature had no significant differences (*P* > 0.05, Wilcoxon rank-sum test) between diarrheic and healthy calves. Only the fecal score value showed significant (*P* < 0.05; Wilcoxon rank-sum test) difference between diarrheal and healthy calves.

### Gut microbiome community in diarrheic and healthy calves

Here, a total of 15 diarrheal and 14 healthy fecal samples met the library requirements for shotgun metagenomic sequencing. The results showed that a total of 2.16 Gb of raw reads were generated, of which 0.94 and 1.21 Gb belonged to DSM and HSM groups, respectively (see Table S4 at https://doi.org/10.7910/DVN/UKE1CC). After quality filtering and removing host genome sequences, 2.15 Gb of clean data was retained. Subsequently, a total of 2,699,417 contigs at the scaffold level were generated using the *de novo* assembly (see Table S5 at https://doi.org/10.7910/DVN/UKE1CC), with a mean N50 length of 7335.89 bp. Furthermore, a total of 2,816,110 non-redundant genes were predicted, with a mean protein length of 234.35 and 211.72 bp in the DSM and HSM groups, respectively (see Table S6 at https://doi.org/10.7910/DVN/UKE1CC).

**Fig 1:**
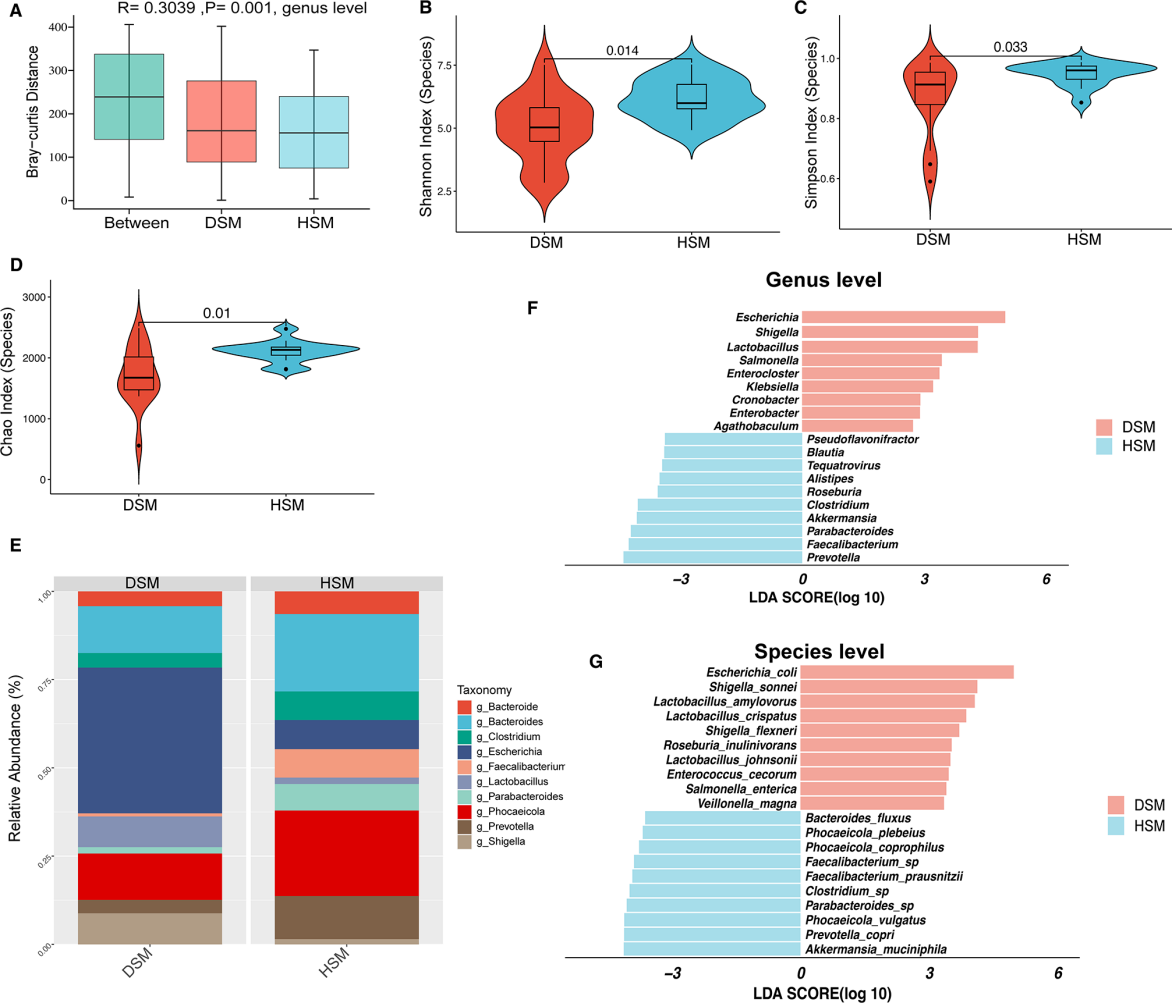
Gut microbiome community in diarrheic and healthy Simmental calves. (**A**) ANOSIM analysis displays the difference in the structure of bacterial community between diarrheic and healthy groups. (**B**) Comparisons of Shannon index of gut microbiota at the species level between diarrheic and healthy groups. (**C**) Comparisons of Simpson’s index of gut microbiota at the species level between diarrheic and healthy groups. (**D**) Comparisons of Chao index of gut microbiota at the species level between diarrheic and healthy groups. (**E**) Bar chart representing the microbiota compositions at the genus level between groups. (**F**) LEfSe analysis (*P* < 0.05 and LDA > 2.5) shows the microbiota compositions at the genus level between groups. (**G**) LEfSe analysis (*P* < 0.05 and LDA > 2.5) shows the microbiota compositions at the species level between groups.

The ANOSIM analysis (*R* = 0.3039, *P* = 0.001) revealed significant differences in the relative abundance of the gut microbiota at the genus level between the two calf groups under study (Fig. 1A). The diarrheic calves showed a lower diversity (Shannon index, Simpson’s index, and Chao) of gut microbiota at the species level compared to the healthy calves (Fig. 1B, C, and D).

To characterize the differences in the composition of the gut microbial of diarrheic calves, we compared the microbial taxonomy between DSM and HSM groups. The top 3 dominant phyla in the DSM group were Pseudomonadota (38.40%), Bacillota (31.57%), and Bacteroidota (22.48%); whereas the Bacillota (42.18%) was the most dominant phyla in HSM group, followed by Bacteroidota (35.88%) and Pseudomonadota (9.73%) (see Fig. S1 at https://doi.org/10.7910/DVN/UKE1CC). The *Escherichia* and *Bacteroides* were the most abundant genera in the DSM group, accounting for 26.71% and 8.58% of the total bacterial community, respectively (Fig. 1E). On the other hand, the most abundant genera in HSM calves were *Phocaeicola*, representing 14.29% of the total microbiome (Fig. 1E). Furthermore, we found that nine genera, including *Escherichia*, *Shigella*, *Lactobacillus*, *Salmonella*, *Enterocloster, Klebsiella, Cronobacter, Enterobacter,* and *Agathobaculum*, were abundant in the DSM calves (*P* < 0.05 and LDA > 2; Fig. 1F). At the species level, the relative abundances of *Escherichia coli* and *Shigella sonnei* were significantly (*P* < 0.05) higher in the DSM calves than HSM calves (Fig. 1G).

### Functional comparison of gut microbiome between diarrheic and healthy calves

**Fig 2:**
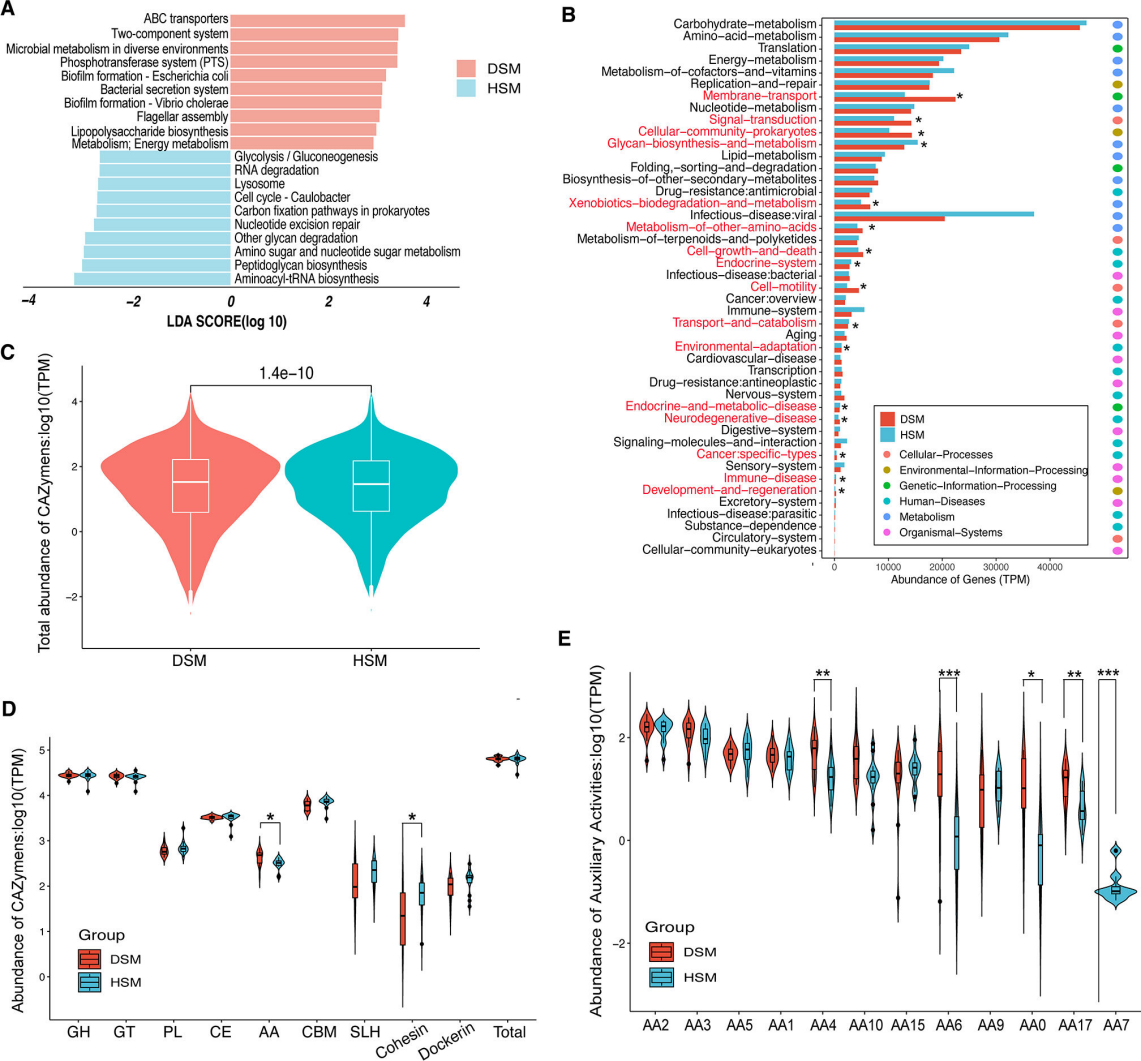
Gut microbial function analysis between diarrheic and healthy Simmental calves. (**A**) Difference of KEGG pathway abundance by LEfSe (*P* < 0.05 and LDA > 2.5). (**B**) Comparison of main KEGG function. The red color means significantly more abundant in the gut microbiome of diarrheic Simmental calves, and green color indicates significantly more abundant in the gut microbiome of healthy Simmental calves. (**C**) Total abundance comparison of CAZymes between groups. (**D**) Abundance comparison of CAZymes between groups. (**E**) Abundance comparison of the auxiliary activities (AA), polysaccharide lyases (PL), and glycoside hydrolase (GH). *, *P*-value < 0.05; **, *P*-value < 0.01; ***, *P*-value < 0.001.

We initially predicted the gene functions of the gut microbiome based on the KEGG database. The most abundant genes in the microbiomes of the DSM group were associated with ABC transporters, two-component regulatory system, microbial metabolism in diverse environment, phosphotransferase system (PTS), and biofilm formation-*Escherichia coli* (Fig. 2A). In contrast, aminoacyl-tRNA biosynthesis was the most abundant pathway in the gut microbiomes of the HSM group (Fig. 2A). The majority of genes enriched in membrane transport were abundant (*P* < 0.05) in the gut microbiomes of diarrheic calves compared to healthy individuals (Fig. 2B), followed by the signal transduction, cellular community prokaryotes, and xenobiotics biodegradation and metabolism.

Furthermore, we explored CAZymes activity in the gut microbiome based on CAZy database. These CAZymes were clustered into six classes: auxiliary activities (AA), carbohydrate-binding modules, carbohydrate esterases, glycoside hydrolases, glycosyl transferases, and polysaccharide lyases. Total genes of CAZymes showed significant differences (*P* < 0.05) in abundance between DSM and HSM groups (Fig. 2C). Notably, we found that these genes encoding AAs and cohesin exhibited a significant (*P* < 0.05) difference between DSM and HSM groups (Fig. 2D). Among them, the AA0, AA4, AA6, AA7, and AA17 in AA family were significantly more abundant in the DSM gut microbiome (Fig. 2E).

### Antibiotic resistance genes of gut microbiome in diarrheic calves

**Fig 3:**
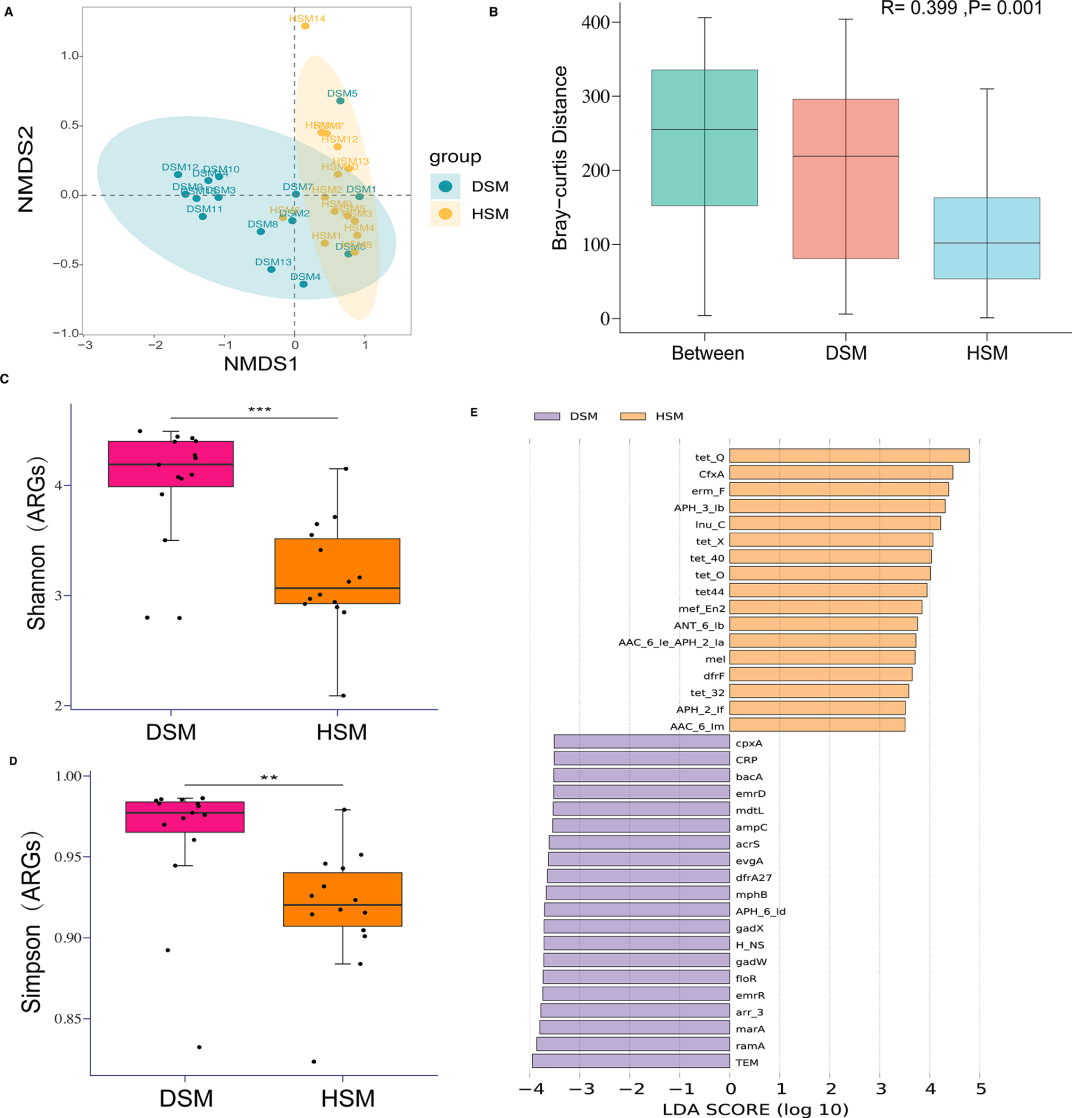
Antimicrobial resistance gene analysis of gut microbiome between diarrheic and healthy Simmental calves. (**A**) DMDS analysis displays the resistance difference of bacterial community between diarrhea and healthy groups. (**B**) ANOSIM analysis of ARGs displays the resistance difference of bacterial community between diarrhea and healthy groups. (**C**) Comparisons of Shannon index of ARGs between diarrheic and healthy groups. (**D**) Comparisons of Simpson’s index of ARGs between diarrheic and healthy groups. (**E**) Difference in the antimicrobial resistance of gut microbiome between groups based on LEfSe analysis (*P* < 0.05 and LDA > 2.5). **, P-value < 0.01; ***, *P*-value < 0.001.

CARD database was used to identify the ARGs of gut microbiome in diarrheic calves, generating a total of 175 ARG subtypes. NMDS2 analysis based on the ARGs profile showed that the abundance difference in ARG between DSM and HSM groups was evident (Fig. 3A). ANOSIM analysis also showed a clear separation between the two groups based on the ARGs abundance (*R* = 0.399, *P* = 0.001; Fig. 3B). Both Shannon and Simpson’s indexes demonstrated that the diarrheic calves had higher (*P* < 0.05) diversity of ARGs than that of the healthy calves (Fig. 3C and D).

To uncover the abundance differences in ARGs between DSM and HSM groups, the LEfSe analysis revealed that a total of 20 ARG subtypes were significantly more abundant in the guts of diarrheic calves compared to the control group (Fig. 3E). Among them, a total of 13 ARGs were predicted to be associated with the resistance mechanism of antibiotic efflux based on the CARD database, followed by the 4 ARGs for antibiotic inactivation, as depicted in Table S7 at https://doi.org/10.7910/DVN/UKE1CC.

### Virulence factor genes of gut microbiome in diarrheic calves

**Fig 4:**
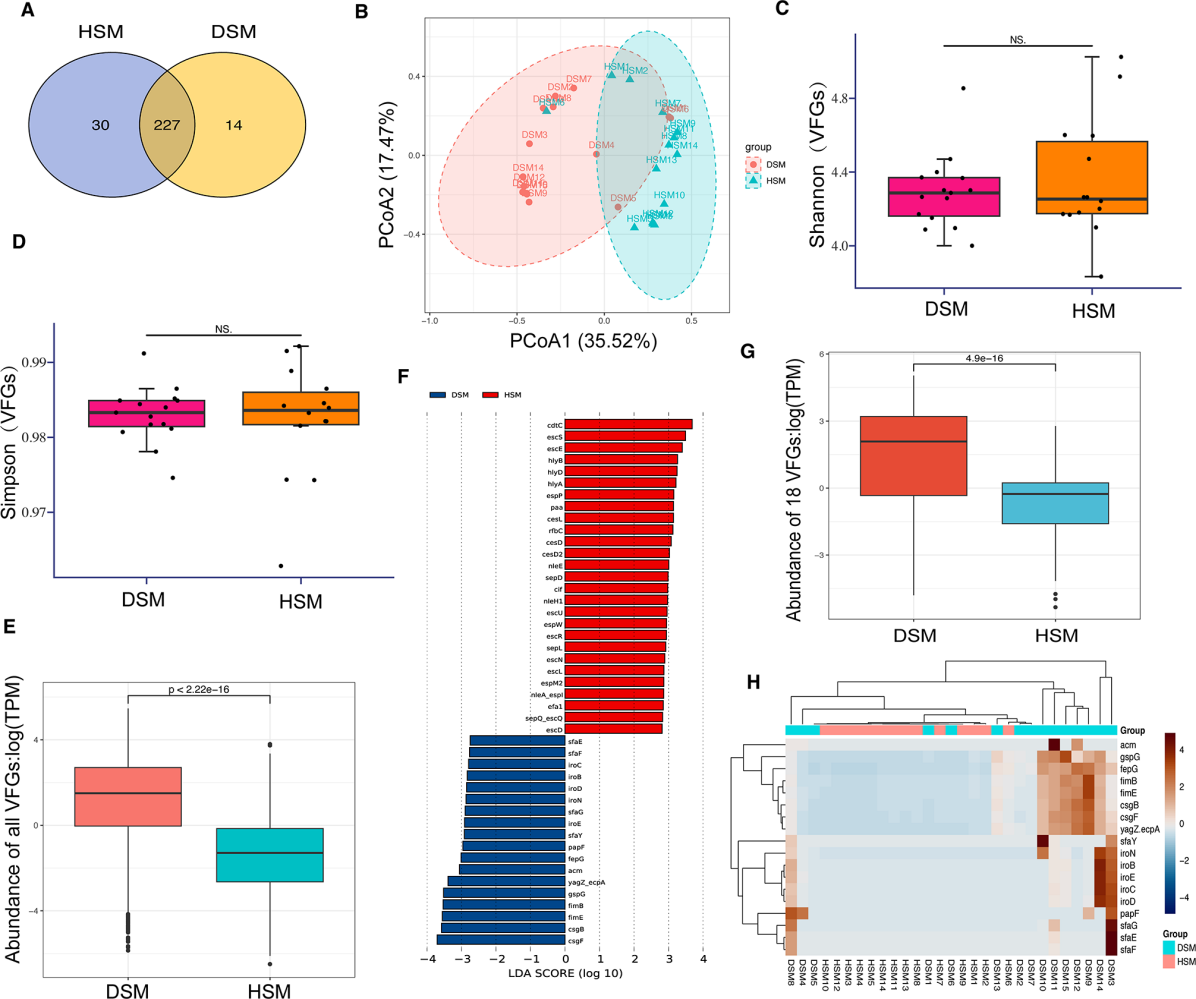
Virulence factors gene analysis of gut microbiome between diarrheic and healthy Simmental calves. (**A**) Venn analysis of VFGs between two groups. (**B**) PCoA analysis for diarrheic and healthy Simmental calves based on VFGs. (**C**) Comparisons of Shannon index of VFGs between diarrheic and healthy groups. (**D**) Comparisons of Simpson’s index of VFGs between diarrheic and healthy groups. (**E**) Comparisons of the abundances of all VFGs between two groups. (**F**) Difference of VFGs abundance between diarrheic and healthy groups by LEfSe (*P* < 0.05 and LDA > 2.5) analysis. (**G**) Comparisons of the abundances of 18 VFGs between two groups. (**H**) Heatmap plot displays the abundance distribution of 18 VFGs across all samples. NS indcates no significant difference.

To identify the VFGs of the gut microbiome in diarrheic calves, the microbial genes were analyzed against the VFDB database. A total of 271 VFGs were detected, of which 227 VFGs were common between the DSM and HSM groups (Fig. 4A). PCoA analysis based on the VFGs abundance revealed that the PCoA1 and PCoA2 explained 35.52% and 17.47% of the variability, respectively, demonstrating that the virulence difference between DSM and HSM groups was almost completely separated (Fig. 4B). No significant difference in the diversity of VFGs between DSM and HSM calves was detected by the Shannon and Simpson’s index (Fig. 4C and D, respectively). However, significant (*P* < 0.05) difference in the abundance of VFGs between the two studied groups was observed (Fig. 4E).

To investigate the abundance differences in VFGs between the diarrheic and healthy calves, LEfSe analysis showed that a total of 27 VFGs were significantly different in abundance between DSM and HSM groups (Fig. 4F). Differentially expressed VFGs had differences (*P* < 0.0001) in abundance between the diarrheic and healthy calves (Fig. 4G). Notably, eight VFGs, including acm, gspG, fepG, fimB, fimE, csgB, csgF, and yagZ/ecpA, were highly expressed abundance in the DSM compared to HSM group (Fig. 4H), indicating that these VFGs might play vital role in the occurrence of diarrhea.

### Correlations between microbial communities and ARGs or VFGs

**Fig 5:**
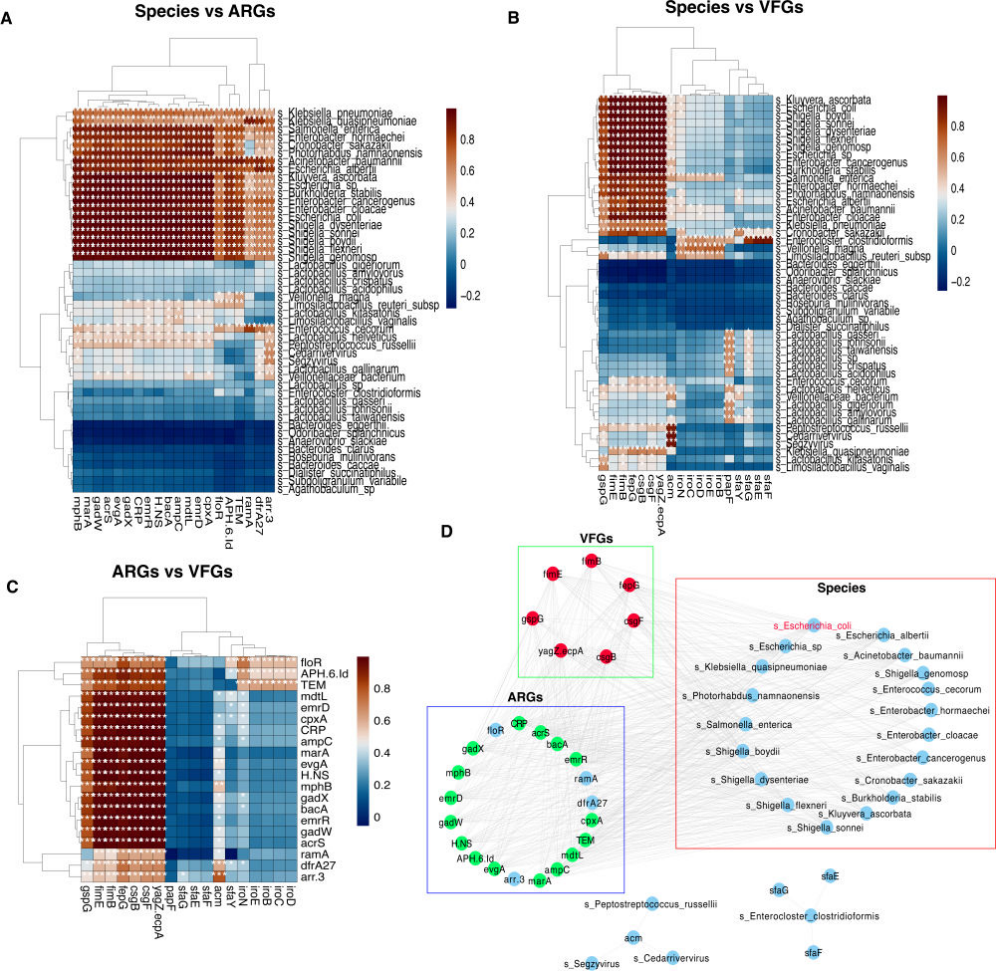
Correlation analysis between ARGs, VFGs, and microbial taxa. (**A**) Correlation between ARGs and microbial taxa. (**B**) Correlation between VFGs and microbial taxa. (**C**) Correlation between VFGs and ARGs. (**D**) Co-occurrence patterns among the ARGs, VFGs, and *Escherichia coli* based on their correlation coefficient of >0.8.

To screen the relationship between ARGs or VFGs and microbial taxa in diarrheic calves, we performed the Pearson correlation analysis to construct the co-occurrence network. The results demonstrated that a total of 48 bacterial genera displayed significant (*P* < 0.0001) correlation with 20 ARGs (correlation coefficient > 0.8; Fig. 5A). *E. coli* revealed strong significant (*P* < 0.0001) correlation coefficient of >0.80 with 16 ARGs, including evgA, emrR, emrD, APH (6)-Id, H-NS, gadX, gadW, mphB, CRP, bacA, cpxA, acrS, marA, TEM-1, mdtL, and ampC1.

Moreover, we predicted the co-occurrence patterns between VFGs and microbial taxa in diarrheic calves, showing that 48 bacterial genera had a strong significant correlation with 18 VFGs (correlation coefficient >0.8; Fig. 5B). Notably, we observed that the *E. coli* showed a very strong significant (*P* < 0.0001) correlation coefficient of >0.80 with 18 VFGs, including iroC, iroD, iroE, gspG, fepG, fimE, sfaY, sfaE, sfaF, sfaG, iroN, iroB, csgF, csgB, papF, acm, yagZ/ecpA, and fimB.

Correlation analysis between ARGs and VFGs revealed that 20 ARGs had strong significant correlation coefficients of >0.8 with 15 VFGs (Fig. 5C). The 20 ARGs were floR, APH (6)-Id, TEM, mdtL, emrD, cpxA, CRP, ampC1, marA, evgA, H-NS, mphB, gadX, bacA, emrR, gadW, acrS, ramA, dfrA27, and arr-3. The 15 VFGs were gspG, fimE, fimB, fepG, csgB, csgF, yagZ/ecpA, sfaG, acm, sfaY, iroN, iroE, iroB, iroC, and iroD.

To clarify the relationship between *E. coli* and ARGs or VFGs, we constructed the interaction network based on their correlation coefficients using Cytoscape software. The results showed that *E. coli* shared the same interaction network with 16 ARGs and 7 VFGs (Fig. 5C). The ARGs contained evgA, emrR, emrD, gadX, CRP, bacA, cpxA, mdtL, ampC1, APH (6)-Id, H-NS, gadW, marA, mphB, acrS, and TEM-1. While the VFGs consisted of gspG, fepG, fimE, csgF, csgB, yagZ/ecpA, and fimB. These results indicate that these ARGs and VFGs may play a vital role in the pathogenesis of *E. coli*.

### Key metabolites in diarrheic calves caused by *E. coli* associated with ARGs and VFGs

**Fig 6:**
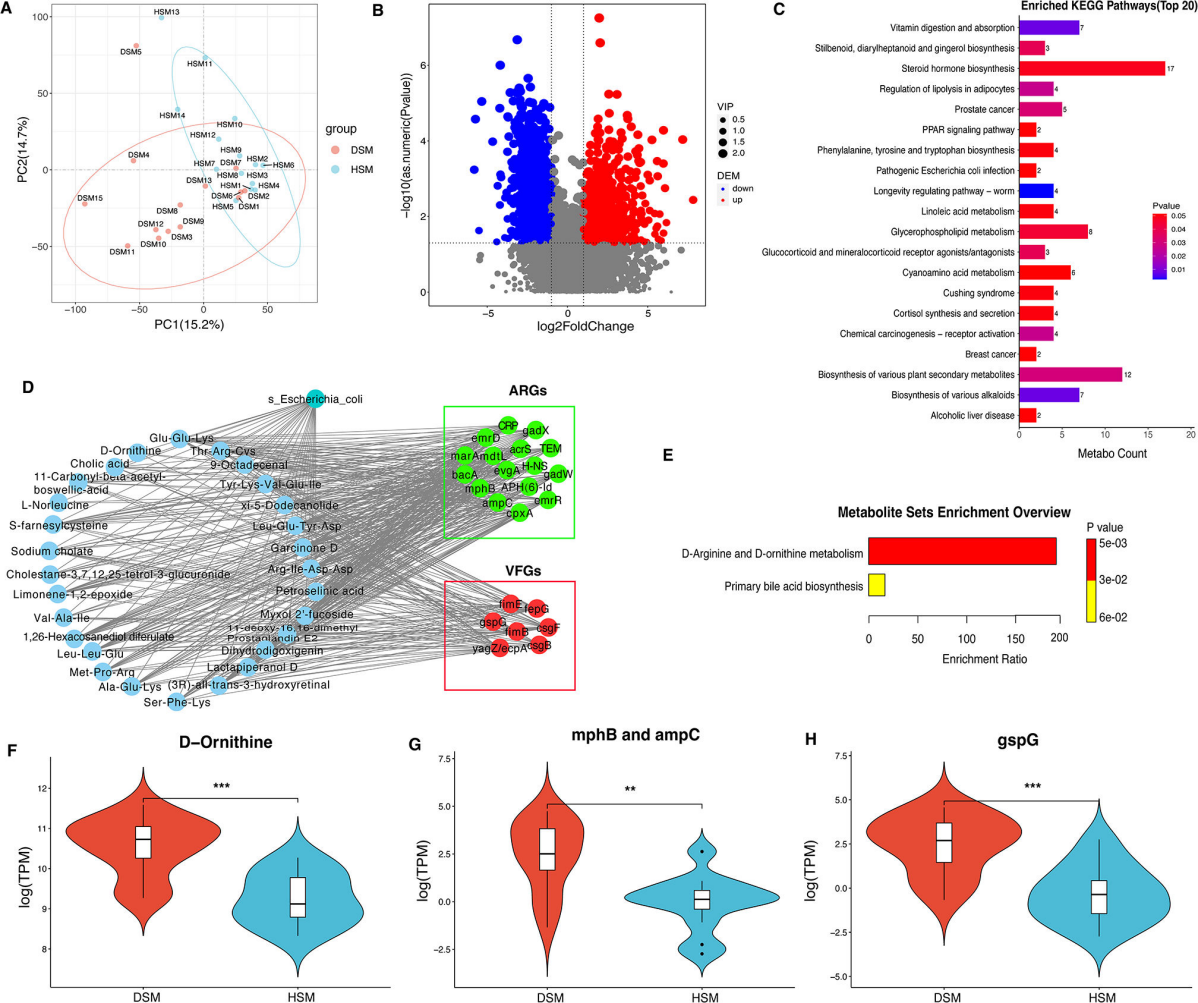
Identification of differentially expressed metabolites related to diarrhea and its correlation with ARGs, VFGs, and microbial taxa. (**A**) PCA analysis for diarrheic and healthy Simmental calves based on metabolites. (**B**) Volcano plot displayed the differentially expressed metabolites between diarrheic and healthy Simmental calves. (**C**) KEGG enrichment analysis of differentially expressed metabolites. (**D**) Co-occurrence patterns among the DEMs, ARGs, VFGs, and *E. coli* based on their correlation coefficient of >0.8 and *P*-value of <0.05. (**E**) KEGG enrichment analysis of DEMs associated with the ARGs, VFGs, and/or *E. coli*. (**F**) Comparisons of the abundances of D-ornithine between two groups. (**G**) Comparisons of the abundances of mphB and ampC between two groups. (**H**) Comparisons of the abundances of gspG between two groups. *, *P*-value < 0.05; **, *P*-value < 0.01; ***, *P*-value < 0.001.

Evidence revealed that the metabolic changes were associated with the incidence of diarrhea (57). We, therefore, used fecal samples for metabonomic analysis to determine the metabolic changes in diarrheic calves. Here, a total of 11,092 and 7,421 metabolites were identified for the positive and negative ion models, respectively. Furthermore, a total of 15,713 unique metabolites were obtained, and the detail information on these metabolites was listed in Table S8 at https://doi.org/10.7910/DVN/UKE1CC. PCA analysis of these metabolites showed that the studied samples had good quality and system stability (Fig. 6A). Moreover, a total of 2,405 DEMs were identified using the significance analysis with VIP > 1, fold change > 2, and *P*-values < 0.05, of which 575 metabolites were upregulated and 1,829 downregulated (Fig. 6B). KEGG analysis of DEMs showed that steroid hormone biosynthesis was the most significant pathway for enrichment (Fig. 6C). Moreover, correlation analysis revealed that 28 DEMs in the diarrhea calves caused by *E. coli* had strong significant correlation coefficients of >0.8 with 16 ARGs and 7 VFGs (Fig. 6D). The 28 DEMs were sodium cholate, Ala-Glu-Lys, Glu-Glu-Lys, Leu-Leu-Glu, Met-Pro-Arg, Ser-Phe-Lys, dihydrodigoxigenin, 9-octadecenal, 11-deoxy-16,16-dimethyl prostaglandin E2, garcinone D, limonene-1,2-epoxide, xi-5-dodecanolide, 1,26-hexacosanediol diferulate, (3R)-all-trans-3-hydroxyretinal, myxol 2*'*-fucoside, Arg-Ile-Asp-Asp, Leu-Glu-Tyr-Asp, Thr-Arg-Cys, Tyr-Lys-Val-Glu-Ile, Val-Ala-Ile, cholestane-3,7,12,25-tetrol-3-glucuronide, cholic acid, acetyl-11-keto-beta-boswellic acid, L-norleucine, lactapiperanol D, petroselinic acid, S-farnesylcysteine, and D-ornithine. Notably, D-ornithine was found to be enriched into the D-arginine and D-ornithine metabolism (Fig. 6E). The relative abundance of D-ornithine was found to be higher in the diarrheic calves compared to the healthy ones (Fig. 6F). Additionally, D-ornithine had a strong correlation with two ARGs and one VFGs. The relative abundance analysis showed that abundances of these ARGs (Fig. 6G) and VFGs (Fig. 6H) both were higher in the diarrheic calves compared to the healthy ones.

### Phylotyping and serotyping for *Escherichia coli*

**Fig 7:**
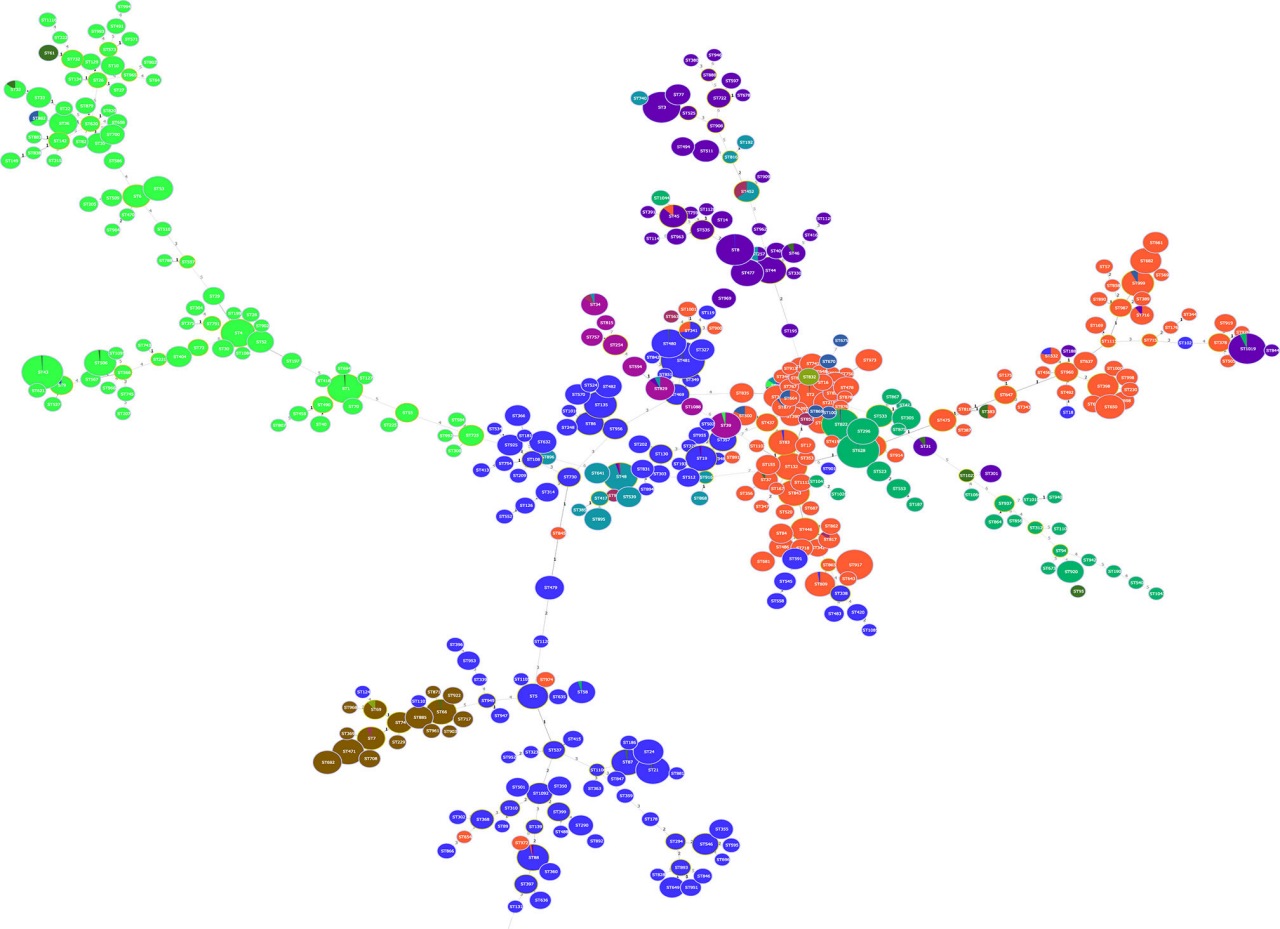
Phylogroup analysis of *Escherichia coli* genome based on their MLST profiles. Note: blue indicates the phylogroup B1, orange indicates the phylogroup A, green indicates phylogroup B2, purple indicates phylogroup D, cyan sky indicates phylogroup E, brown indicates phylogroup C, bright purple indicates phylogroup G, pink indicates phylogroup F, and dark sapphire indicates phylogroup unknown.

Considering the importance of phylogroup or serotype of *E. coli* genome on antimicrobial resistance and virulence factors, we performed the phylotyping and serogrouping for 10,076 *E. coli* genomes. The results revealed that a total of 6,456 *E. coli* genomes had the detection signals of phylotyping and serogrouping (see Table S9 at https://doi.org/10.7910/DVN/UKE1CC). These genomes can be classified into eight phylogroups, as depicted in Fig. 7. Among them, the phylogroup B1 had 1,746 genomes, accounting for 27.04%, followed by phylogroup A (*n* = 1,544, 23.92%), phylogroup B2 (*n* = 947, 14.67%), phylogroup D (*n* = 836, 12.95%), phylogroup E (*n* = 793, 12.28%), phylogroup C (*n* = 210, 3.25%), phylogroup G (*n* = 156, 2.42%), and phylogroup F (*n* = 144, 2.23%). For the diarrheal Simmental calves, three individuals belonged to phylogroup B1 and O45 serotypes (Table 1). The phylogroups A, B2, C, and F for *E. coli* ST were detected in the studied individuals. Moreover, 10 ARGs [emrR, CRP, ampC1, mphB, acrS, cpxA, evgA, marA, H-NS, and APH (6)-Id] and 3 VFGs (fimB, fimE, and yagZ/ecpA) showed high correlation with the *E. coli* from phylogroup B1.

**TABLE 1:** Phylotype, serotype, ARGs, and VFGs of *E. coli* genomes in diarrheic Simmental calves^*a*^^,*b*^

Name	Phylogroups	ST	Serotype	ARGs	VFGs
DSM1	B1	87	O45:H25	mdtP, mdtO, mdtN, eptA, **emrR**, emrA, emrB, **CRP**, mdtE, mdtF, tolC, acrA, acrB, ampH, **ampC1**, acrD, **mphB**, msbA, **acrS**, acrE, acrF, **cpxA**, pmrF, evgS, **evgA**, emrK, emrY, baeR, baeS, mdtC, mdtB, mdtA, mdtG, mdtH, yojI, **marA**	**fimB**, **fimE**, fimI, fimC, fimD, fimG, yagW/ecpD, yagX/ecpC, yagY/ecpB, **yagZ/ecpA**, iucA, iucB, iucC, iucD, iutA, f17d-D, f17d-C, afaA-VIII, afaB-VIII, afaC-VIII, afaD-VIII, cdtB
DSM2	B1	87	O45/O4:H31	**CRP**, eptA, mdtN, mdtO, mdtP, **acrS**, acrE, acrF, **mphB**, acrD, **ampC1**, msbA, acrB, **acrA**, mdtH, mdtG, pmrF, tolC, **emrR**, emrA, emrB, mdtF, mdtE, **cpxA**, emrY, emrK, **evgA**, evgS, ampH, **H-NS**, baeS, mdtC, mdtB, mdtA, **marA**	fyuA, ybtE, ybtT, irp1, ybtA, ybtP, ybtQ, ybtX, ybtS, fimD, **yagZ/ecpA**, yagY/ecpB, yagX/ecpC, yagW/ecpD, f17d-D, f17d-C, iucA, iucB, iucC, iucD, iutA, fimC, fimI, cdtB
DSM3	U	21	O83:H42	MCR-1, mdtG, oqxA, oqxB, tet(B), emrK, CMY-59, **CRP**, **mphB**	sfaC, sfaB, sfaD, sfaE, sfaF, sfaG, sfaY, afaF-VII, iroC, iroD, iroE, iroN, chuT, chuU, chuV, fepB, **fimB**, fimI, fimG, entB, papK, papF, fimG
DSM4	F	731	O83:H42	mdtH, **H-NS**, oqxB, oqxA, mdtG, APH(3')-Ia, acrD, emrA, **emrR**, **CRP**, **marA, mphB**	chuT, chuU, chuV, fimG, fimD, yagW/ecpD, **fimB, fimE**, yagY/ecpB, fimC, fimI
DSM5	U	7	NA^*c*^	mdtE	**FimE**, yagY/ecpB, afaF-VII
DSM6	U	69	O45:H9	**bacA**, acrF, acrE, **acrS**, **cpxA**, mdtF, mdtE, eptA, tolC, ugd, **CRP**, **emrR**, mdtP, mdtO, mdtN, mdtA, emrB, ampH, acrB, baeR, baeS, **evgA**, emrK, yojI, pmrF	cdtB, f17d-D, f17d-C, iutA, iucD, iucC, iucB, iucA, yagX/ecpC, fimI, fimC, ybtP, **fimB**, ybtS
DSM7	B1	357	O23:H16	emrY, emrK, **evgA**, evgS, **ampC1**, acrD, mdtF, mdtE, **CRP**, **H-NS**, tolC, mdtG, mdtH, pmrF, ampH, acrB, acrA, acrE, acrF, baeR, baeS, mdtC, mdtA, msbA, marA, **emrR**, emrA, emrB, eptA, mdtO, mdtP, cpxA, tet(B), **APH (6)-Id**, APH(3*''*)-Ib	fimG, fimD, fimC, fimI, **fimE**, ompA, **yagZ/ecpA**, yagY/ecpB, yagX/ecpC, yagW/ecpD, f17d-G, f17d-C, f17d-D, f17d-A, fyuA, ybtP, ybtA, afaF-VII, ybtS, iucB
DSM8	B2	731	O83:H9	MCR-1, oqxB, oqxA, mdtG, tet(B), emrK, **TEM-1**, CMY-59, kdpE, **mphB**	sfaC, sfaB, sfaD, sfaE, sfaF, sfaG, sfaY, iroC, iroD, iroE, iroN, chuV, chuU, chuT, astA, fepB, **yagZ/ecpA**, yagY/ecpB, ykgK/ecpR, yagW/ecpD, fimF
DSM9	U	2	O174/O83:H28	MCR-1, msbA, **H-NS**, acrA, acrB, tolC, mdtH, pmrF, emrY, emrK, **evgA**, evgS, **APH (6)-Id**, APH(3*''*)-Ib, sul2, mdtP, mdtO, mdtN, **CRP**, mphA, **ampC1**, ampH, emrB, oqxB, oqxA, dfrA1, catB3, AAC(6')-Ib7, yojI, **cpxA**, **mphB**, baeR, acrE, **acrS**, CMY-59, CTX-M-55	**yagZ/ecpA**, yagY/ecpB, yagX/ecpC, yagW/ecpD, chuV, chuU, chuT, entB, fimC, fimI, astA, **fimB**, yagY/ecpB
DSM10	A	388	O160:H14	mdtG, mdtH, tolC, acrB, acrA, mdtP, mdtO, **mphB**, acrD, pmrF, yojI, emrB, emrA, **emrR**, mdtA, eptA, evgS, **evgA**, emrK, emrY, **marA**, mdtE, mdtF, acrF, acrE, **acrS**, cpxA, msbA, **H-NS, CRP**, ampH, oqxA, oqxB, AAC (3)-IId, baeR, baeS, mphA, **TEM-1**	fimD, fimI, **fimE**, f17d-C, f17d-D, **yagZ/ecpA**, yagY/ecpB, yagX/ecpC, yagW/ecpD, afaF-VII, papX
DSM11	U	21	O83:H37	MCR-1, oqxB, oqxA, mdtA, AAC (3)-IId, eptA, **TEM-1**, pmrF, **CRP**, mdtE, CMY-59, **mphB**	iroC, iroD, iroE, iroN, chuT, chuU, chuV, astA, **fimE, fimB**, yagW/ecpD, ykgK/ecpR, fimI, sfaD, sfaY, fimH, fimI, sfaG, fimF
DSM12	C	692	O8:H9	eptA, evgS, **evgA**, emrK, emrY, msbA, acrB, acrA, cpxA, tolC, **acrS**, acrE, acrF, **bacA**, pmrF, acrD, mdtG, **CRP**, mdtF, mdtE, baeR, baeS, mdtC, mdtB, mdtH, emrB, **emrR**, **H-NS**, CMY-59, marA, yojI, ampH, floR, tet(B), oqxA, **TEM-1**	fyuA, ybtE, ybtT, ybtU, irp1, irp2, ybtA, ybtP, ybtQ, ybtX, ybtS, afaA-VIII, afaB-VIII, afaC-VIII, afaD-VIII, **yagZ/ecpA**, yagY/ecpB, yagX/ecpC, yagW/ecpD, f17d-D, f17d-C, fimG, fimD, fimC, fimI, **fimE**
DSM13	C	7	O21/O45:H9	pmrF, yojI, msbA, acrF, acrE, **acrS**, eptA, acrD, **ampC1, CRP, H-NS, acrA**, acrB, mdtH, mdtG, mdtP, mdtO, mdtN, **bacA**, tolC, marA, **cpxA**, emrY, emrK, **evgA**, evgS, mdtF, mdtE, emrR, emrB, mdtA, mdtB, mdtC, baeS, baeR, ampH	fyuA, ybtE, ybtT, ybtU, irp1, irp2, ybtA, ybtP, ybtQ, ybtX, ybtS, yagW/ecpD, yagX/ecpC, yagY/ecpB, **yagZ/ecpA**, f17d-D, f17d-C, cdtB, iutA, iucD, iucC, iucB, iucA, fimD, fimC, fimI, f17d-A
DSM14	U	31	O9/O117/ O107:H30	marA, baeR, baeS, mdtC, ampH, mdtG, acrA, acrB, **CRP**, msbA, oqxB, oqxA, mphA, **evgA**, emrK, acrE, **acrS**, kdpE, yojI, **emrR**, mdtE, CMY-59, **H-NS, gadX**	ybtS, ybtX, ybtQ, ybtP, ybtA, irp2, irp1, ybtU, ybtT, ybtE, fyuA, iroN, iroE, iroD, iroC, iroB, f17d-C, f17d-D, hlyA, hlyB, hlyD, gspH, shuS, shuA, shuT, shuX, chuU, chuV, espX2, ykgK/ecpR, **yagZ/ecpA**, espY3, espY2, espX4, espY1, fimI, gspM
DSM15	U	46	O7/O83:H10	emrE, oqxB, oqxA, QepA4, tet(B), baeR, CMY-59, **acrS**, APH(3')-Ia, catI, **H-NS**, mdtG	ybtS, ybtX, ybtQ, ybtP, ybtA, irp2, irp1, ybtU, ybtT, ybtE, fyuA, f17d-C, f17d-D, kpsD, iroN, iroE, iroD, iroC, afaA-VIII, afaB-VIII, afaC-VIII, afaD-VIII, papC, papD, papJ, papK, espX2, chuT, espY2, chuU, gspH, afaE-VIII, ykgK/ecpR, astA, gspM, afaF-VII, shuX, fimI, **fimB**

^*a*^ U indicates the phylogroup unknown.

^*b*^ Bold fonts indicate ARGs or VFGs that are highly associated with calf diarrhea.

^*c*^ NA indicates the serotype unknown

## DISCUSSION

In the present study, the microbiomes and resistomes of purebred Simmental calves suffering from diarrhea were explored based on shotgun metagenomic sequencing. Importantly, we found that several bacterial species varied between the diarrheic and healthy calves. The Pseudomonadota (38.40%)*,* Bacillota (31.57%), and Bacteroidota (22.48%) were the three most dominant phyla accounting for a major proportion of the gut microbiota in the diarrheic calves, which is quite similar to the relative abundances already reported in diarrheic animals (58–60). Significant differences in the abundance of nine genera, including *Escherichia*, *Shigella*, *Lactobacillus*, *Salmonella*, *Enterocloster, Klebsiella, Cronobacter, Enterobacter,* and *Agathobaculum,* were observed in the guts of diarrheic calves. Considering the species, *Escherichia coli* and *Shigella sonnei* were more abundant in the DSM than HSM groups. Previous studies have demonstrated associations between *Escherichia* (61)*, Shigella* (62)*, Lactobacillus* (63), *Salmonella* (64)*, Enterobacter* (65), and *Klebsiella* (66) and diarrhea in animals. In addition, several distinct bacterial taxa were identified at the species level as being different between the healthy calves and those calves suffering from diarrheal disease in our study. It is noteworthy that the *Escherichia coli* and *Shigella sonnei*, as pathogens, were significantly (*P* < 0.05) different in the relative abundance of diarrheic calves compared to healthy calves. It is well known that both *E. coli* and *Shigella sonnei* are the pathogenic bacteria and associated with increased incidence of diarrhea in farm animals (67–69) and humans (70). These findings showed that *E. coli* played a vital role in the occurrence of diarrhea.

Gut microbiota plays an important role in shaping the health status of animals through alteration of the host’s metabolic functions (71). In the present study, we observed differences at the functional gene levels between diarrheic and healthy calves. These microbial genes were mainly involved in the ABC transporters, two-component system, microbial metabolism in diverse environment, PTS, and biofilm formation *Escherichia coli*. Evidence showed that ABC transporters were increasingly recognized for their ability to modulate the absorption, distribution, metabolism, secretion, and toxicity of xenobiotics (72). Interestingly, our data showed that the abundance of microbial genes in the xenobiotics biodegradation and metabolism pathway was increased in the diarrheal calves compared to healthy individuals. Additionally, the two-component systems were reported to be the predominant way bacteria adapt to changing environments and modulate their fitness in various niches (73–75). These results indicate that the microbial genes may play a vital role in the adaptability to xenobiotic toxicity. Furthermore, we observed significant differences in the relative abundances of CAZymes between diarrheic and healthy calves, particularly with regard to some genes encoding AAs. It has been reported that AAs are involved in the catabolism of carbohydrates (76). This could imply that the bacterial genes encoding AAs may impact the gut microbiome metabolism in individuals affected by diarrhea. Cheng et al. (77) also highlighted specific carbohydrates that play a crucial role in human diseases by regulating the imbalanced intestinal microbiota.

Widespread antibiotic use causes antibiotic selection pressure, resulting in potential emergence of antibiotic-resistant bacteria and reduction of microbiota diversity, thereby accelerating the rise of AMR levels (78–80). It is well known that coexisting microbes can produce antibiotics or bacteriocins in response to the competition for nutrients in the intestinal digesta (81–83). Here, we observed that the diarrheal calves had higher abundance and diversity of ARGs compared to healthy calves. This might be partially explained by the differences in the ability of bacterial species to adapt to changes in nutritional habits, diets, and environmental conditions on farms as well as selection pressures exerted by antibiotics (84). Similar findings have been reported by other studies (85, 86). Importantly, significant differences in the richness and the abundance of ARGs between diarrheal and healthy calves were observed. These ARGs contained the floR, APH (6)-Id, TEM, mdtL, emrD, cpxA, CRP, ampC, marA, evgA, H-NS, mphB, gadX, bacA, emrR, gadW, acrS, ramA, dfrA27, and arr-3. According to resistance mechanism analysis of CARD database, a total of 13 ARGs, including the floR, mdtL, emrD, cpxA, CRP, marA, evgA, H-NS, gadX, emrR, gadW, acrS, and ramA, were the antibiotic efflux, followed by 4 ARGs (TEM, ampC, mphB, and arr-3) and APH (6)-Id for antibiotic inactivation, bacA for antibiotic target alteration, and dfrA27 for antibiotic target replacement. These results indicate that the resistance mechanisms of these ARGs in the gut microbiome are mainly involved in the antibiotic efflux.

Co-occurrence pattern showed that a total of 48 bacterial genera displayed significant (*P* < 0.0001) correlation with 20 ARGs (correlation coefficients >0.8). Importantly, these bacteria have been demonstrated to play essential roles in the drug resistance through the resistance mechanism of antibiotic efflux (87–89). Notably, our analysis showed that *Escherichia coli* had highly significant (*P* < 0.0001) correlation coefficients of >0.8 with 16 ARGs, which were mainly involved in the antibiotic efflux. These findings are in agreement with earlier studies demonstrating that *E. coli* harbored the largest number of ARGs, showing multidrug resistance in a wide variety of environments (90–92). Moreover, for these *E. coli*-mediated ARGs, they had higher abundance in the diarrheal calves compared to healthy individuals. These results point out that the development of antimicrobial resistance in diarrhea calves may be related to the use of maternal antibiotics, which are passed through the mother’s colostrum.

Virulence factors of a bacterium are molecules secreted, membrane-associated or cytosolic agents that help the bacteria to colonize their hosts (93). Bacterial pathogens use their secretion system to directly incapacitate target cells and enhance their virulence (94). In the present study, we found that 18 VFGs, including the iroC, iroD, iroE, gspG, fepG, fimE, sfaY, sfaE, sfaF, sfaG, iroN, iroB, csgF, csgB, papF, acm, yagZ/ecpA, and fimB, were significantly enriched in the diarrheic calves (*P* < 0.05 and LDA >2.5) compared to the healthy individuals. In particular, co-occurrence patterns showed that *E. coli* had strong positive correlation coefficients of >0.8 with the aforementioned VFGs. Previous studies shown that both fimB and fimE were responsible for controlling the expression of type I fimbriae in *E. coli* (95–97). Swasthi et al. (98) reported that curli were functional amyloids present on the outer membrane of *E. coli*, while csgB and csgF were required for curli assembly (98). Four genes (sfaY, sfaE, sfaF, and sfaG) were known to play a vital role in the adhesion of S-fimbriae in the pathogenesis of *E. coli* meningitis (99). Evidence showed that five iro genes (iroC, iroD, iroE, iroN, and iroB) were required for the virulence of Avian pathogenic *E. coli* (100). Additionally, it is well documented that fepG was essential for ferrienterobactin transport in *E. coli* (101), while papF played various functional roles in pilus biogenesis of *E. coli* (102). These results indicate that these VFGs may be involved in various functions of *E. coli* pathogenicity.

Interestingly, we found that *E. coli* interacted with 16 ARGs and 7 VFGs. These ARGs contained evgA, emrR, emrD, gadX, CRP, bacA, cpxA, mdtL, ampC, APH (6)-Id, H-NS, gadW, marA, mphB, acrS, and TEM-1. It has been noted that these ARGs mainly use antibiotic efflux mechanisms as resistance mechanisms. This means that those calves with diarrhea exhibit drug-resistant properties in response to *E. coli* infection, which subsequently provide reliable basis for veterinary personnel to prevent and treat diarrhea. Moreover, the seven VFGs detected were gspG, fepG, fimE, fimB, csgF, csgB, and yagZ/ecpA. Evidence showed that gspG (103) and yagZ/ecpA (104) were associated with the type IV pilus and common pilus structural subunits of *E.coli,* respectively. Based on our previous discussion, it can be inferred that these VFGs might play a role in the pili function, curli assembly, and ferrienterobactin transport of *E. coli*. In other words, the results obtained point out that both ARGs and VFGs may play a crucial role in the pathogenesis of *E. coli*. Moreover, we found that 28 DEMs in the diarrhea calve caused by the *E. coli* were significantly associated with the 16 ARGs and 7 VFGs. These DEMs were sodium cholate, Ala-Glu-Lys, Glu-Glu-Lys, Leu-Leu-Glu, Met-Pro-Arg, Ser-Phe-Lys, dihydrodigoxigenin, 9-octadecenal, 11-deoxy-16,16-dimethyl prostaglandin E2, garcinone D, limonene-1,2-epoxide, xi-5-dodecanolide, 1,26-hexacosanediol diferulate, (3R)-all-trans-3-hydroxyretinal, myxol 2*'*-fucoside, Arg-Ile-Asp-Asp, Leu-Glu-Tyr-Asp, Thr-Arg-Cys, Tyr-Lys-Val-Glu-Ile, Val-Ala-Ile, cholestane-3,7,12,25-tetrol-3-glucuronide, cholic acid, acetyl-11-keto-beta-boswellic acid, L-norleucine, lactapiperanol D, petroselinic acid, S-farnesylcysteine, and D-ornithine. Evidence showed that petroselinic acid played an important role in the biofilm formation and virulence factor production (105). The acetyl-11-keto-beta-boswellic acid is known to exhibit potent anti-inflammatory properties *in vitro* and *in vivo* (106). The 11-deoxy-16,16-dimethyl prostaglandin E2 was confirmed to protect against oxidative stress (107), which is responsible for a variety of degenerative processes including diarrhea (108). Limonene-1,2-epoxide is one derivative of limonene that is associated with diarrhea (109). In addition, 10 compounds (Ala-Glu-Lys, Glu-Glu-Lys, Leu-Leu-Glu, Met-Pro-Arg, Ser-Phe-Lys, Arg-Ile-Asp-Asp, Leu-Glu-Tyr-Asp, Thr-Arg-Cys, Tyr-Lys-Val-Glu-Ile, and Val-Ala-Ile) related to amino acids were found, and previous studies have shown that amino acids played vital role in intestinal inflammation (110) and had adverse gastrointestinal effects (111). Moreover, we found that the 28 DEMs were mainly enriched in the D-arginine and D-ornithine metabolism and primary bile acid biosynthesis. Evidence showed that the D-arginine and D-ornithine metabolism play a vital role in intimately participating in permeability and adaptive responses of the gut (112), while the bile acid biosynthesis was mainly involved in the bile acid diarrhea (113). These results suggest that these metabolites may play a role in the occurrence of diarrhea, possibly though multiple biological pathways or functions.

*E. coli* isolates are known to be a vast diversity in terms of clonal lineages due to the transition between commensalism, mutualism, and pathogenicity, depending on the selection pressures and niche-specific constraints or adaptations experienced by the host genomes (114). In this study, we demonstrated that the currently investigated *E. coli* genomes constructed from 15 samples consisted of 8 major phylogroups, which is similar to previous studies (115–117). For the diarrheal Simmental calves, we observed that three individuals had *E. coli* genomes from the phylogroup B1, while seven individuals had *E. coli* genomes from unknown phylogroups. Coura et al. (118) found that the phylogroups B1 and E were associated with *E. coli* isolated from cattle, while phylogroups B2 and D were associated with *E. coli* collected from water buffalo (118). Recently, Özgen et al. (119) reported that the phylogenetic group B1 has been observed in the case of bovine abortions (119). Notably, Aguirre-Sánchez et al. (120) mentioned that the phylogroup B1 for *E. coli* isolated from cattle located in Mexico demonstrated a significant relationship with food sources (120). Moreover, we found that 10 ARGs (emrR, CRP, ampC1, mphB, acrS, cpxA, evgA, marA, H-NS, and APH (6)-Id) and 3 VFGs (fimB, fimE, and yagZ/ecpA) showed strong correlations with the *E. coli* from phylogroup B1. Therefore, it can be inferred that the ARGs are mainly related to antibiotic efflux, while the VFGs are correlated to the pilus function of *E. coli*. These results imply that these ARGs and VFGs may play a vital role in the occurrence of diarrhea incidences caused by *E. coli* in the Simmental calves.

### Conclusions

In summary, our study concluded that the relative abundances of fecal microbiota in the diarrhea calves displayed drastic changes between diarrhea and healthy calves. Diarrheic Simmental calves exhibited significant differences in abundance of antibiotic resistance and virulence factor genes. Importantly, our findings revealed that *E. coli* had a strong significant positive correlation with most of the ARGs and VFGs. The ARGs in diarrhea calves were mainly involved in antibiotic efflux, while these VFGs were associated with VFGs with pili function, curli assembly, and ferrienterobactin transport of *E. coli*. Metabolomics analysis showed that differentially expressed metabolites in Simmental calves with diarrhea displayed a high correlation with the aforementioned ARGs and VFGs. Our study provides crucial insights into the gut microbiome and ARGs of neonatal calves affected with diarrhea.

## Data Availability

Raw sequencing data of shotgun metagenomics sequencing have been deposited into the China National GeneBank DataBase (CNGBdb; https://db.cngb.org) and NCBI Sequence Read Archive (SRA) database under accession numbers CNP0005069 and PRJNA843354, respectively.
